# Effect of Semaglutide Versus Placebo on Heart Failure With Preserved Ejection Fraction in Obese Patients: A Systematic Review

**DOI:** 10.7759/cureus.85250

**Published:** 2025-06-02

**Authors:** Carlos Alberto Umaña Mejia, Claudia Victoria Sañudo Soto, Juan Robalino, Mayra Hernández, Myriam Bertha Santos Bretón, Edwin A Garcia-Vasquez, Paola Rocha, Luis Miguel Palacios Brambila, Sergio Morales, Miguel Romo, Jaqueline L Castillo, Mauricio Montelongo Quevedo, Jose R Flores Valdés

**Affiliations:** 1 General Medicine, Universidad Autonoma De Guadalajara, Guadalajara, MEX; 2 General Medicine, Universidad Autónoma De Durango, Sinaloa, MEX; 3 General Medicine, Universidad Técnica De Ambato, Ambato, ECU; 4 General Medicine, Universidad Anáhuac Querétaro, Querétaro, MEX; 5 General Medicine, Universidad De Las Américas, Puebla, MEX; 6 General Medicine, Universidad San Martin Porres, Chiclayo, PER; 7 Internal Medicine, Hospital General Dr. Alfredo Pumarejo, Matamoros, MEX; 8 Cardiology, Universidad Westhill, Mexico City, MEX; 9 Cardiology, Hospital Civil De Guadalajara Fray Antonio Alcalde, Guadalajara, MEX; 10 Internal Medicine, Instituto Mexicano De Seguridad Social, Aguascalientes, MEX; 11 General Medicine, Oncology Consultants, Houston, USA

**Keywords:** biomarkers, heart failure, obesity, preserved ventricular ejection fraction, semaglutide

## Abstract

Heart failure (HF) is a major global health concern and can be classified into different phenotypes based on left ventricular ejection fraction (LVEF), including heart failure with preserved ejection fraction (HFpEF), reduced ejection fraction, and mildly reduced ejection fraction. This systematic review aims to determine the effect of semaglutide compared to placebo in obese patients with HFpEF. Following PRISMA 2020 guidelines, relevant studies published from January 2021 to August 2024 were identified through searches in PubMed and Science Direct. Randomized clinical trials (RCTs), cohort studies, and case-control studies were considered; however, only two randomized controlled trials (RCTs) and one retrospective cohort ultimately met the inclusion criteria, encompassing a total of 1463 participants with HFpEF and obesity. The risk of bias was evaluated utilizing the Cochrane risk of bias tool for RCTs and the Newcastle-Ottawa Scale (NOS) for the retrospective cohort. In each study, participants were divided into two groups receiving either semaglutide or placebo. The findings after a 52-week follow-up showed that treatment with semaglutide 2.4 mg once weekly resulted in a significant reduction in biomarkers associated with HF. Specifically, baseline C-reactive protein (CRP) values decreased by 43% and 42% in the RCTs and 37% in the retrospective cohort. NT-proBNP levels declined by 20.90% and 23.20% in the RCTs and 15.80% in the cohort, compared to the placebo group. The reductions in CRP and NT-proBNP are clinically relevant, as elevated levels of these biomarkers are associated with worse outcomes in HFpEF. In addition, participants in the semaglutide group experienced a reduction in body weight ranging from 9% to 13% across the three studies, while those in the placebo group showed weight loss between 2% and 7% across the studies. Functional improvement was also observed, with the Kansas City Cardiomyopathy Questionnaire-Clinical Summary Score (KCCQ-CSS) increasing by 13 to 16 points in the semaglutide group compared to the placebo. These results suggest that semaglutide may be a promising treatment for HFpEF in obese patients, offering not only significant weight loss but also improvements in biomarkers and quality of life. Therefore, semaglutide appears to provide potential cardiovascular and metabolic benefits in addition to its established weight-reducing effects, although findings should be interpreted with caution given the limited number of included studies.

## Introduction and background

Heart failure (HF) is characterized by the heart's inability to pump blood effectively to meet the body's demands or by the need to elevate filling pressures to compensate for this deficiency [1]. Heart failure with preserved ejection fraction (HFpEF) is a clinical syndrome in patients with current or prior symptoms of HF with a left ventricular ejection fraction (LVEF) ≥50% and evidence of cardiac dysfunction as the cause of symptoms [2]. Key risk factors for HFpEF include age, hypertension, obesity, diabetes, and atrial fibrillation. The prevalence of HFpEF is rising and is projected to surpass that of HFrEF in the near future [3]. This increase in HFpEF cases is largely due to the aging population and the growing prevalence of risk factors, particularly obesity, metabolic syndrome, and type 2 diabetes, which are closely linked to the development of the condition. The total burden of HFpEF is projected to become the dominant HF subtype, affecting approximately one in 10 adults during their lifetime [4]. 

Patients with HFpEF are hospitalized approximately 1.4 times per year and have an annual mortality rate of approximately 15% [4]. For patients with HFpEF, the goals of treatment are to reduce HF symptoms, increase functional status, and reduce the risk of hospital admission. The majority of patients with HFpEF are obese; defined as a body mass index of 30 or more, and they are distinguished by a notably elevated burden of HF-related symptoms and physical limitations, in addition to unfavorable hemodynamics and an increased risk of unfavorable cardiac events [5]. Currently, it is unknown whether the use of pharmacotherapies specifically targeting obesity can reduce symptoms and physical limitations, and improve exercise function in these patients [6]. Obesity contributes to myocardial dysfunction and HF risk through multiple interrelated mechanisms. These include hemodynamic alterations, neurohormonal activation, and the endocrine and paracrine effects of adipose tissue. Additionally, ectopic fat deposition and lipotoxicity play critical roles. Collectively, these processes promote concentric left ventricular (LV) remodeling and predominantly increase the risk for HFpEF [7].

The Food and Drug Administration (FDA) supports the idea that an HF treatment may be approved based only on improvements in physical function and a decrease in symptoms. Evidence from structured patient interviews suggests that people living with HF place as much importance on symptom relief and enhanced physical ability as they do on survival. So far, there have been few treatments that effectively address the issues patients consider vital to their quality of life, underscoring a major unmet need [6]. Many trials on HFpEF have focused on measuring the long-term consequences of hypertension; however, obesity is also an independent risk factor for the development of HF, the effects of which must be studied [8]. Semaglutide is a glucagon-like peptide-1 (GLP-1) receptor agonist, which acts on the incretin hormone system. It stimulates beta-pancreatic cells to release insulin in hyperglycemic states and also reduces gastric emptying and as such decreases food intake [9].

The importance of this systematic review lies in the fact that HF is associated with considerable functional limitations, increased morbidity, and a lower quality of life, which highlights the need to accelerate advances in therapeutic strategies for patients with this disease [10]. According to some recent studies, semaglutide could offer a promising therapeutic option for managing HFpEF in individuals with obesity. For this reason and because there is not yet a study that synthesizes this evidence together, the objective of this study is to integrate results on whether semaglutide, unlike placebo, could cause weight loss, improvements in Kansas City Cardiomyopathy Questionnaire-Clinical Summary Score (KCCQ-CSS), 6-minute walking distance (6MWD), and biomarkers in obese patients with HFpEF. 

## Review

Methods 

This review was conducted following the 2020 guidelines for Preferred Reporting Items for Systematic Reviews and Meta-Analyses (PRISMA) and was guided by evidence-based medicine principles to ensure a thorough and methodical approach [11,12].

*Search Methods * 

Stringent selection parameters were defined to ensure the inclusion of only high quality studies. Exclusion criteria were strictly enforced to preserve both the relevance and integrity of the data analyzed.

A comprehensive literature search was carried out using PubMed and ScienceDirect databases. The search strategy incorporated a combination of Medical Subject Headings (MeSH) and relevant free-text keywords aligned with the research objectives. The selection of articles was systematically documented using a PRISMA flow diagram. This rigorous process supported the development of a consistent dataset, thereby enhancing the reliability and accuracy of the findings. Keywords utilized in the search included “semaglutide,” “heart failure,” “obesity,” and “clinical trial.”

Selection criteria 

Types of Participants 

This study specified criteria that included individuals aged 18 or older, both sexes, patients with HFpEF, and obesity (body mass index of at least 30). The exclusion criteria for participants in the reviewed studies were those involving pediatric populations, patients under 18 years, pregnant patients, patients with HFrEF, or a patient-reported significant change in body weight within the days prior to screening. 

*Types of Intervention* 

This systematic review focuses on evaluating the effect of semaglutide in obese patients with HFpEF. The intervention includes subcutaneous administration of semaglutide at a dose of 2.4 mg. The control group receives a matching placebo. 

*Types of Studies* 

In our investigation of the effects of semaglutide versus placebo on HFpEF in obese patients, we conducted a thorough review of relevant studies published in English and Spanish between January 2021 and August 2024. Studies not published in English or Spanish were excluded, as these are not the primary languages of the author. It is acknowledged that this may introduce a potential risk of bias due to the narrowing of the search. We evaluated studies that met the prespecified inclusion criteria, including randomized clinical trials (RCTs), cohort studies, and case-control studies reporting the effects of semaglutide use in obese patients with HFpEF. To ensure research quality, we excluded case reports, case series, cross-sectional studies, dissertations, letters to the editor, and comment publications. 

*Types of Outcomes * 

In our systematic review, we included all studies meeting the inclusion criteria to evaluate the effects of semaglutide versus placebo on HFpEF in obese patients. The primary outcome of the study is to investigate the effects of semaglutide 2.4 mg compared with placebo, looking for the following: 1) change in KCCQ-CSS and 2) percent change in body weight. Secondary outcomes included assessing the effects of semaglutide on change in 6MWD, change in the long-transformed C-reactive protein (CRP), and N-terminal Pro-b-type natriuretic peptide (NT-proBNP).

Selection of Studies, Data Extraction, and Screening

Rayyan [13] was used by two reviewers (MH and CS) to screen titles and abstracts, while a third reviewer (MP) independently assessed the relevance of the studies based on predefined criteria. Following this, two more reviewers (SM and MR) performed a detailed full-text analysis, selecting studies according to the same criteria. Discrepancies at this stage were resolved through consensus and assistance from a third reviewer (EGV). The retrieved studies were thoroughly evaluated against the inclusion and exclusion criteria for relevance.

Data was extracted from the studies selected for inclusion as follows: (a) general information (author, title, publication date, language, and age of participants); (b) study characteristics (study design); (c) intervention (the number of cases/controls or cohort groups, inclusion criteria used, and description of it); and (d) outcome data (baseline and follow‐up measure). 

*Data Evaluation: Assessment of Risk of Bias * 

Our evaluation adhered to the guidelines provided in the Cochrane Handbook for Systematic Reviews of Interventions [14]. For cohort studies, we employed the Newcastle-Ottawa Scale (NOS) [15], and for RCTs, we used the Cochrane risk of bias tool [16,17]. Two independent reviewers (YT and MS) assessed the risk of bias for each study, applying the criteria and guidelines specific to the respective tools. Any disagreements between reviewers were resolved through discussion with a third blinded reviewer (MP). 

Results 

A detailed review of the literature was done to discover the effect of semaglutide on obese patients with HFpEF. Specifically, we focused on how a semaglutide dose of 2.4 once a week could impact KCCQ-CSS score, walk distance, and biomarker levels (CRP and NT-proBNP) in a time of one year. 

A key aspect of a systematic review is the meticulous identification and selection of relevant studies from a vast array of literature. Our search strategy began with a broad query across databases, resulting in 195 articles. After careful de-duplication, this number was reduced to 166. Following a systematic screening of titles and abstracts, 138 articles were excluded, and 23 were selected for full-text review. Ultimately, this process led to the inclusion of three high-quality studies that satisfied our stringent criteria. This included two RCTs and one retrospective cohort.

**Figure 1:**
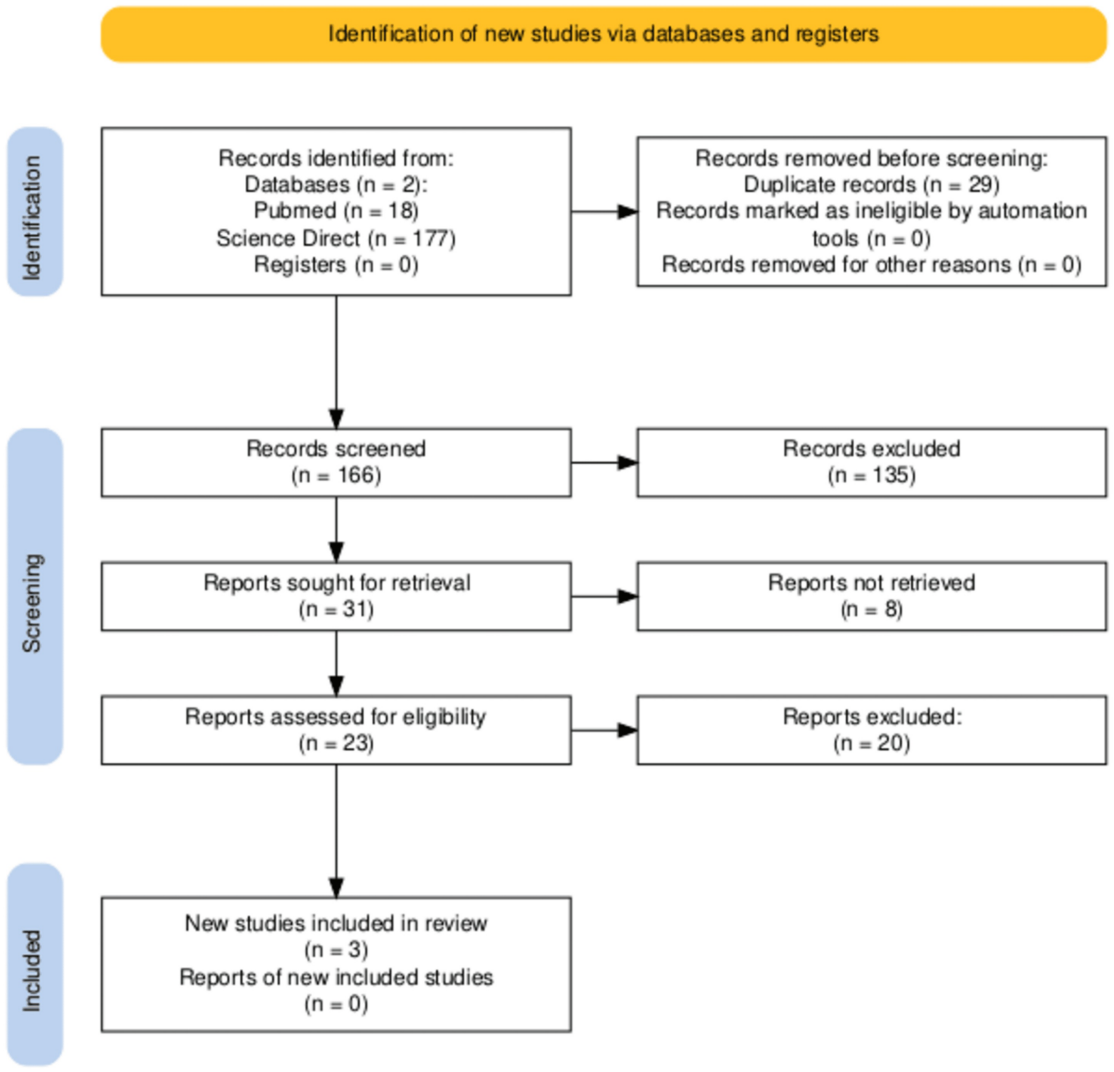
Flow diagram of bibliographic search

Figure 1 provides a clear visual representation of the study selection process, based on the structure of the PRISMA [11] flow diagram. This step-by-step diagram enhances transparency in our screening procedure, leading to the final selection of studies. The findings from the included studies are presented in Table 1 [10].

**Table 1:** Summary of general clinical outcomes CRP, c-reactive protein; NT-proBNP, N-terminal Pro-b-type natriuretic peptide; NYHA, New York Heart Association; KCCQ-CSS, Kansas City Cardiomyopathy Questionnaire-Clinical Summary Score; CAD, coronary artery disease

Author, Year	Kosiborod, et al., 2023 [6]	Kosiborod, et al., 2024 [18]	Rehman et al., 2024 [19]
Study type	Randomized Controlled Trial	Randomized Controlled trial	Retrospective Cohort
Total patients in study	529	616	318
Intervention group (semaglutide)	263	310	104
Placebo group	266	306	214
Semaglutide dose	2.4 mg once weekly	2.4 mg once weekly	2.4 mg once weekly
Mean age - intervention group	70 years old (62-75)	69 years old (62-74)	70 years old (62-75)
Mean A=age - placebo group	69 years old (62-75)	70 years old (63-75)	69 years old (63-75)
Sex - intervention group (F/M)	149 female and 114 male	128 female and 182 male	59 female and 45 male
Sex - placebo group (F/M)	148 female and 118 male	145 female and 161 male	113 female and 101 male
Ethnicity - intervention group	African American: 3%, Caucasian: 97%	Caucasian: 81.0%, Asian: 14.5%, African American: 4.2%, Other: 0.3%	Punjabi: 2.9%, Kashmiri: 93.3%
Ethnicity - placebo group	African American: 4.9%, Caucasian: 94.7%	Caucasian: 87.6%, Asian: 10.1%, African American: 1.6%, Other: 0.7%	Punjabi: 3.7%, Kashmiri: 95.3%
Cardiovascular disease history and risk factors - intervention group	Atrial fibrillation: 135, hypertension: 216, CAD: 53, NYHA class II: 183, NYHA class III/IV: 80	Hypertension: 255, atrial fibrillation: 117, CAD: 79, OSA: 25	Atrial fibrillation: 54, hypertension: 82, CAD: 23, NYHA class II: 69, NYHA class III/IV: 35
Cardiovascular disease history and risk factors - placebo group	Atrial fibrillation: 140, hypertension: 217, CAD: 45, NYHA class II: 167, NYHA class III/IV: 98	Hypertension: 271, atrial fibrillation: 126, CAD: 69, obstructive sleep apnea: 28	Atrial fibrillation: 102, hypertension: 163, CAD: 47, NYHA class II: 125, NYHA class III/IV: 89
Biomarkers	CRP and NT-proBNP	CRP and NT-proBNP	CRP, NT-proBNP
Follow-up time	52 weeks	52 weeks	52 weeks
Number of deaths in intervention group	3	6	3
Number of deaths in placebo group	5	10	5
Adverse events in intervention group	Cardiac disorder: 8, atrial fibrillation: 3, infection: 5, gastrointestinal disorder: 9, nervous system disorder: 8, urinary disorder: 7, injury/poisoning/procedural: 4, musculoskeletal events: 5, metabolism or nutrition disorder: 3, hepatobiliary disorder: 4, benign, neoplasm: 1, general disorder at administration site: 1	Cardiac disorder: 23, arrhythmia: 13, coronary artery disorder: 5, heart failure: 4, vascular disorder: 5, infection: 17, gastrointestinal disorder: 5, nervous system disorder: 7, urinary disorder: 2, respiratory event: 6, injury/poisoning/procedural: 11, metabolism or nutrition disorder: 11, musculoskeletal events: 6, neoplasm: 8, general disorder at administration site: 1	Cardiac disorder: 1, infection: 1, gastrointestinal disorder: 1, nervous system disorder: 1, urinary disorder: 1, injury/poisoning/procedural: 1, musculoskeletal events: 1
Adverse events in placebo group	Cardiac disorder: 43, atrial fibrillation: 12, cardiac failure: 13, atrial flutter: 5, congestive cardiac failure: 3, infection: 22, gastrointestinal disorder: 8, nervous system disorder: 7, urinary disorder: 6, respiratory events: 11, injury/poisoning/procedural: 4, musculoskeletal events: 5, metabolism or nutrition disorder: 4, hepatobiliary disorder: 2, neoplasm: 3, general disorder at administration site: 3	Cardiac disorder: 58, arrhythmia: 12, coronary artery disorder: 10, heart failure: 35, vascular disorder: 6, infection: 38, gastrointestinal disorder: 5, nervous system disorder: 7, urinary disorder: 8, respiratory event: 7, injury/poisoning/procedural: 2, metabolic/nutrition disorder: 4, musculoskeletal events: 8, neoplasm: 7, general disorder at administration site: 3	Cardiac disorder: 4, atrial fibrillation: 2, infection: 4, gastrointestinal disorder: 1, nervous system disorder: 1, urinary disorder: 1, respiratory event: 2, injury/poisoning/procedural: 4, musculoskeletal events: 1, metabolic/nutrition disorder: 1, general disorder at administration site: 1
Change in KCCQ-CSS score (baseline to 52 weeks) - intervention group	16.6	13.7	NA
Change in KCCQ-CSS score (baseline to 52 weeks) - placebo group	8.7	6.4	NA
Percentage change in body weight (baseline to 52 weeks) - intervention group	-13.30%	-9.80%	-10.70%
Percentage change in body weight (baseline to 52 weeks) - placebo group	-2.60%	-3.40%	-7.80%
Change in 6-minute walk distance (baseline to 52 weeks) - intervention group	21.5 meters	12.7 meters	18.7 meters
Change in 6-minute walk distance (baseline to 52 weeks) - placebo group	1.2 meters	-1.6 meters	3.6 meters
Percentage change in CRP level (baseline to 52 weeks) - intervention group	-43.50%	-42.00%	-37.20%
Percentage change in CRP level (baseline to 52 weeks) - placebo group	-7.30%	-12.80%	-12.10%
Percentage change in NT-proBNP level (baseline to 52 weeks) - intervention group	-20.90%	-23.20%	-15.80%
Percentage change in NT-proBNP level (baseline to 52 weeks) - placebo group	-5.30%	-4.60%	-6.70%
Key points	Semaglutide led to larger reductions in heart failure symptoms and physical limitations compared to placebo, along with reductions in CRP, NT-proBNP, and improvements in 6-minute walking distance and Kansas City Cardiomyopathy Questionnaire Clinical Summary Score (KCCQ-CSS). Greater weight loss was also observed compared to placebo.	Semaglutide reduced symptoms in patients with HFpEF and type 2 diabetes, improved physical capacity, and enhanced quality of life (as measured by KCCQ-CSS). It significantly reduced NT-proBNP and CRP levels. There was no evidence of an increase in adverse events, despite the intrinsic risk in patients with diabetes who present with comorbidities.	Semaglutide resulted in significant improvement in exercise capacity, increased weight loss, and reductions in CRP and NT-proBNP levels.

**Figure 2:**
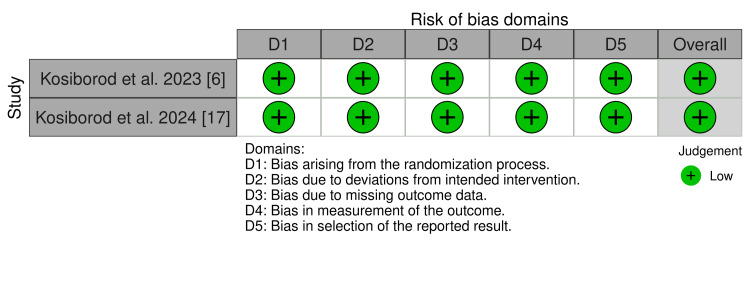
Risk of bias Each article was assessed for risk of bias [15,16]; of the two articles evaluated, both exhibited a low risk of bias [6,17].

**Figure 3:**
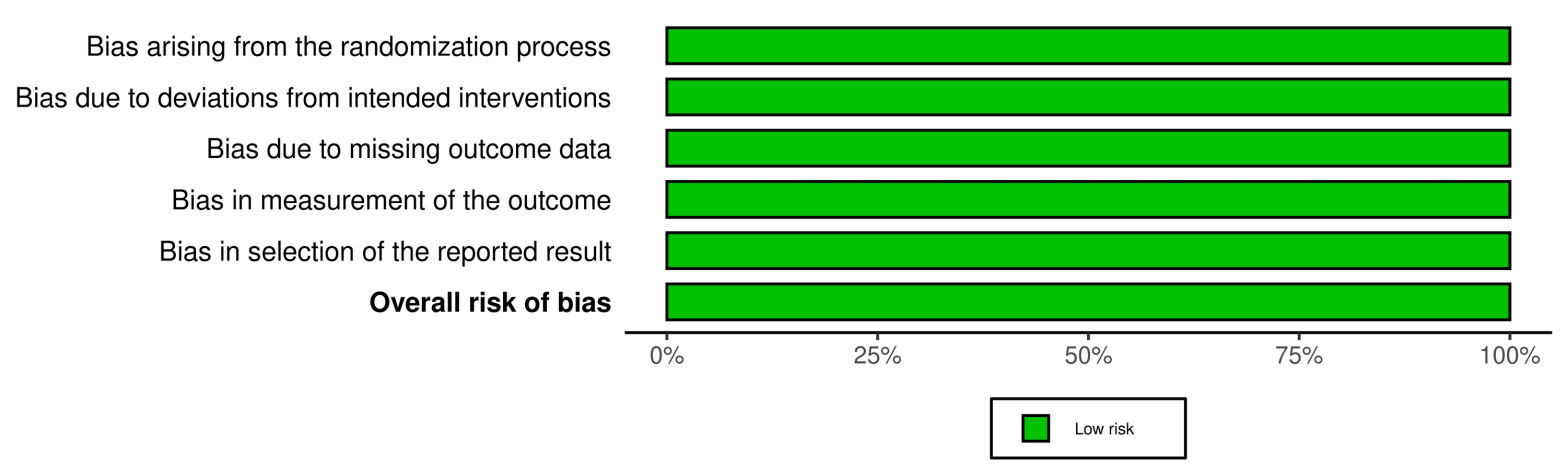
Summary plot for randomized controlled trials This diagram [16,17] illustrates part of the risk of bias assessment, with overall results represented in a single color, green, indicating low risk [6,18].

In a combined analysis of three studies involving 1463 patients with HFpEF and obesity, participants were divided into two groups: the placebo group (assigned with 786 patients) and the semaglutide group (assigned with 677 patients). Participants had a mean age between 69 and 70 years (range: 62-75 years old), with the majority being women. Patients had a common history of cardiovascular diseases such as hypertension and atrial fibrillation, along with risk factors including a New York Heart Association (NYHA) functional classification of II-IV. After a 52-week follow-up, the group treated with semaglutide 2.4 mg once weekly showed significant improvements in heart failure biomarkers. CRP levels decreased by 43% (CI: 0.61 (0.51 to 0.72), p < 0.001) in Kosiborod et al. 2023, 42% (CI: 0.67 (0.55 to 0.80), p < 0.001) in Kosiborod et al. 2024, and 37% (CI: 0.47 (0.32 to 0.62), p < 0.001) in Rehman et al. NT-proBNP levels were reduced by 20.90% and 23.20% in Kosiborod et al. 2023 and 2024, respectively, and by 15.80% (CI: 0.80 (0.56 to 1.14), p = 0.240) in Rehman et al. In contrast, the placebo group showed CRP reductions of only 7% and 12% in RCTs, and 12.10% in a cohort study. NT-proBNP declined by 5.30% and 4.60% in RCTs, and 6.70% in the cohort. Semaglutide also led to significant weight loss: 13.30% (CI: -10.7 (-11.9 to -9.4), p < 0.001) in Kosiborod et al. 2023, 9.80% (CI: -6.4 (-7.6 to -5.2), p < 0.001) in Kosiborod et al. 2024, and 10.7% (CI: -2.9 (-4.1 to -1.7), p < 0.001) in Rehman et al., compared to 2.6%, 3.4%, and 7.8% in placebo groups, respectively. Physical capacity also improved, with increases in 6MWD of 18 to 21 meters and about 12 meters in patients with type 2 diabetes [6,18,19]. KCCQ-CSS scores increased by 13 points (CI: 7.3 (4.1 to 10.4), p < 0.001) and 16 points (CI: 7.8 (4.8 to 10.9), p < 0.001) in two studies [6,18], while Rehman et al. [19] did not assess this measure. The placebo group, by comparison, showed only a 1 to 3 meter increase in 6MWD and a 6 to 8 point improvement in KCCQ-CSS. The risk of bias was assessed as low in the selected RCTs (Figure 2 and Figure 3), and the cohort study was rated as good quality using the NOS (Table 2) [16,17].

**Table 2:** Newcastle-Ottawa tool for cohort studies: risk of bias appraisal The article was assessed for risk of bias and was rated as having good quality [15,19].

Author, year	Study design	Selection	Comparability	Outcome	Total	Subjective evaluation
Rehman et al., 2024 [19]	Cohort study	4	1	3	8	Good quality

Discussion

HFpEF is characterized by impaired ventricular diastolic function, a preserved LVEF (≥50%), LV hypertrophy, and left atrial dilation. These abnormalities lead to significant functional deterioration, especially in individuals with obesity [2]. Given the strong link between HFpEF and obesity, the incidence and prevalence of this condition have increased, making it essential to prioritize managing obesity as a core therapeutic goal [1].

Recent studies suggest that semaglutide, due to its potential benefits in weight loss and improvements in both cardiovascular and metabolic outcomes, could be an effective treatment for patients with HFpEF and obesity. Results show that patients treated with semaglutide at a dose of 2.4 mg for 52 weeks presented a notable reduction in percentage of body weight by 9% to 13%, which is clinically relevant given the strong link between obesity and worsening HF. This weight loss was accompanied by reductions in biomarkers, such as CRP and NT-proBNP. A decrease from baseline CRP values of 37% to 43% and NT-proBNP values of 15% to 23% was observed in the semaglutide group. The anti-inflammatory effect is particularly important as it highlights semaglutide's potential to address the systemic inflammation often present in obesity and HFpEF [6,18,19].

Moreover, semaglutide was associated with improvements in physical function, with patients walking 18-21 additional meters in the 6MWD when compared to placebo [6,18,19]. This improvement in exercise capacity, coupled with an increase of 6 to 8 points on the KCCQ-CSS [6,18], reflects meaningful symptomatic and functional improvements in patients who often experience substantial limitations in daily activities due to HFpEF. While cardiac function was not directly measured via echocardiographic parameters, these functional improvements may indicate favorable cardiovascular effects.

It is crucial to note that the evidence supporting these findings comes from a combination of RCTs and one observational cohort study. The synthesis of results across these study designs requires caution, as RCTs provide higher internal validity, while cohort studies may be more prone to bias and confounding.

Despite these promising results, one key challenge in implementing this treatment is the limited follow-up period of 52 weeks. Given that in the heart, there is an adaptive response to chronic conditions such as HFpEF, it is important to understand if the benefits obtained can be sustained over the long term. It is also important to mention that RCTs included in this review were done in more than 13 countries around different continents increasing the generalizability of the results. From a real-world perspective, the cost, accessibility, and need for subcutaneous administration of semaglutide may influence adherence and limit widespread adoption, particularly in resource-constrained settings. While semaglutide is not currently a standard therapy in HFpEF treatment guidelines, these findings may complement existing management strategies, particularly in patients with coexisting obesity. Continued research may help clarify semaglutide’s role and determine whether it could be integrated into guideline-directed therapy for HFpEF in the future. 

## Conclusions

In conclusion, this systematic review highlights semaglutide as a promising treatment for HFpEF in patients with obesity. Given the high prevalence of obesity in this patient group, effective management of weight is crucial to improving both clinical outcomes and quality of life. The evidence presented in this review demonstrates that semaglutide not only leads to significant weight loss but also results in improvements in functional status as reflected by KCC-QSS scores. Moreover, patients also presented with significant reductions in key biomarkers such as CRP and NT-proBNP. These results are of significant clinical importance as they demonstrate that semaglutide offers substantial cardiovascular and metabolic benefits in addition to its weight-reducing effects. 

However, while the results are encouraging, this systematic review identifies several limitations, such as the lack of studies with follow-up periods beyond 52 weeks and the reliance on a combination of RCTs and observational cohort studies. To establish the long-term safety and efficacy of semaglutide in this population, further RCTs are essential. Additionally, head-to-head comparisons between semaglutide and current guideline-directed therapies for HFpEF are needed to determine its relative efficacy and potential role within existing treatment pathways. Despite these gaps, current evidence supports semaglutide as a valuable treatment option for patients with HFpEF and obesity, and future studies will be crucial to confirm its role in routine clinical care.
